# Posterior Cruciate Ligament Reconstruction in Elite Athletes: A Narrative Review

**DOI:** 10.1007/s43465-026-01794-2

**Published:** 2026-05-07

**Authors:** Darby Amelia Elliott, Chinmay Madhukar Gupte

**Affiliations:** 1 Department of Orthopaedic Surgery and Sports Medicine, London, UK; 2 Imperial College London, Exhibition Road, London, SW7 2AZ UK

**Keywords:** Posterior cruciate ligament, Elite athlete, Multi-ligament knee injury, Reconstruction, Return to play, Dynamic bracing

## Abstract

**Background:**

Posterior cruciate ligament (PCL) injuries are uncommon in sport but may be career-defining for elite athletes. Controversy remains regarding indications for reconstruction, optimal techniques and the role of braced non-operative care. This narrative review summarises evidence on operative and non-operative management of PCL injuries in elite athletes, focusing on return-to-play (RTP), graft selection, reconstruction strategy and elite-specific considerations.

**Methods:**

A narrative review was conducted in PubMed and Embase (inception-April 2025) with reference list screening. Clinical studies reporting return-to-play outcomes after operative or non-operative management of isolated PCL injuries and PCL-based MLKIs in athletic cohorts were prioritised; 51 studies were included, and heterogeneity precluded meta-analysis.

**Results:**

Isolated PCL injuries in elite footballers are rare, whereas PCL-based multi-ligament knee injuries (MLKIs) are more common in collision sports. Early dynamic bracing with quadriceps-focused rehabilitation enables > 90% RTP in selected acute isolated grade II–III tears in high-level athletic cohorts (Level III evidence). For chronic instability or MLKIs, anatomic single-bundle reconstruction remains common, with double-bundle and tibial inlay approaches offering biomechanical but limited clinical advantages. Modern elite MLKI cohorts report 80–90% RTP, though full return-to-performance is less frequent.

**Conclusions:**

Management of elite PCL injuries requires individualised, sport-specific decision-making that balances biological healing with performance demands. High-quality, sport-specific outcome data remain limited.

Level of Evidence

Level V, narrative review.

## Introduction

**Fig. 1:**
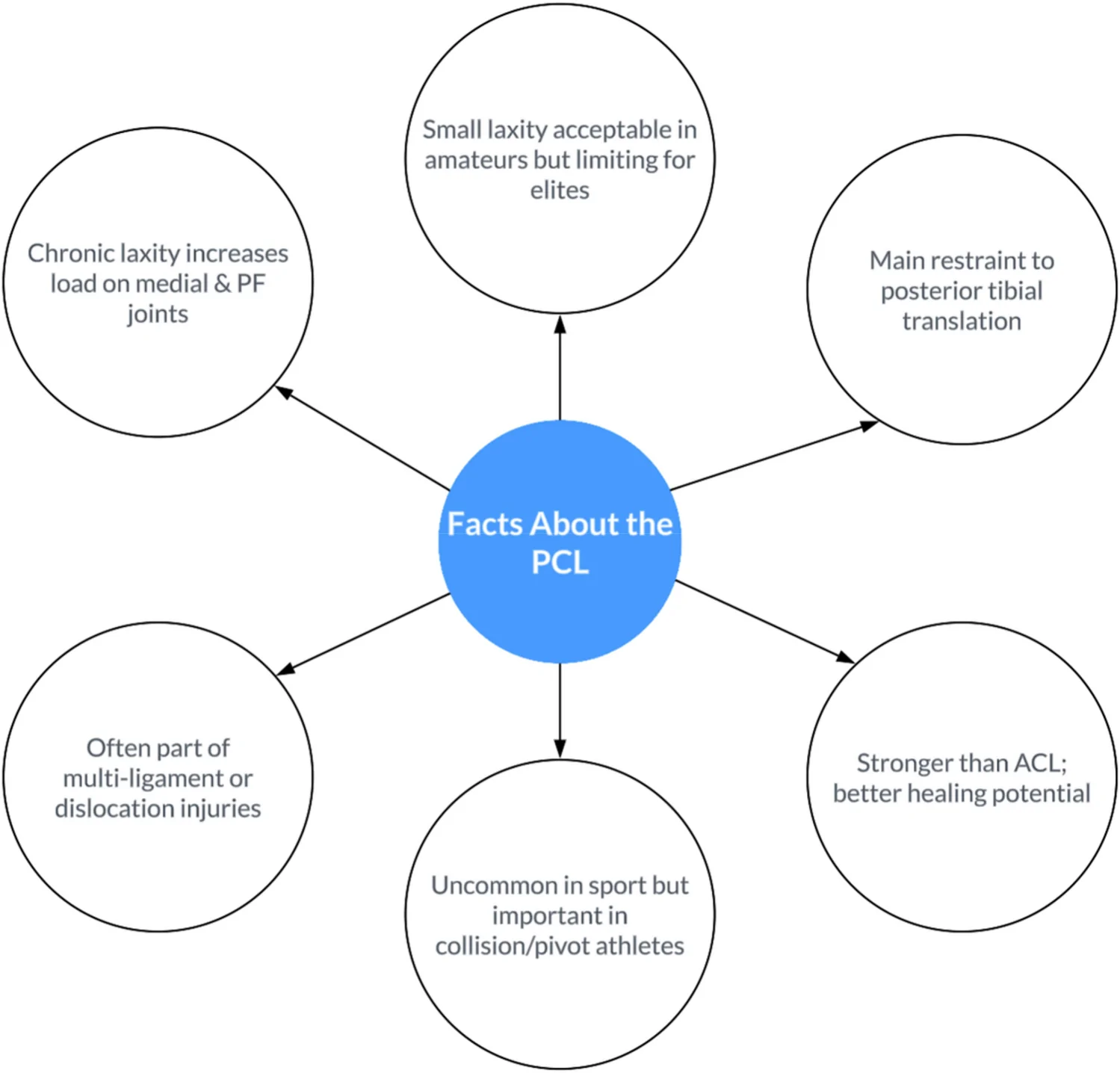
HYPERLINK "sps:id::fig1||locator::gr1||MediaObject::0" Key biomechanical and clinical facts about the posterior cruciate ligament

Posterior cruciate ligament (PCL) injuries represent a minority of ligamentous knee trauma but may be disproportionately important in elite sport, where even small residual instability can impair performance and shorten careers [1]. In comparison with anterior cruciate ligament (ACL) rupture, PCL injuries in professional football and rugby are infrequent, but they are over-represented in multi-ligament knee injuries (MLKIs) and knee dislocations [2]. The PCL also differs biologically from the ACL in possessing greater intrinsic healing potential, which has fuelled longstanding controversy regarding the indications for reconstruction, optimal timing of surgery and the role of dynamic bracing [3]. Although ACL literature provides useful parallels for return-to-play (RTP) decision-making, ACL and PCL injury patterns and healing potential differ biomechanically (Fig. 1).

**Fig. 2:**
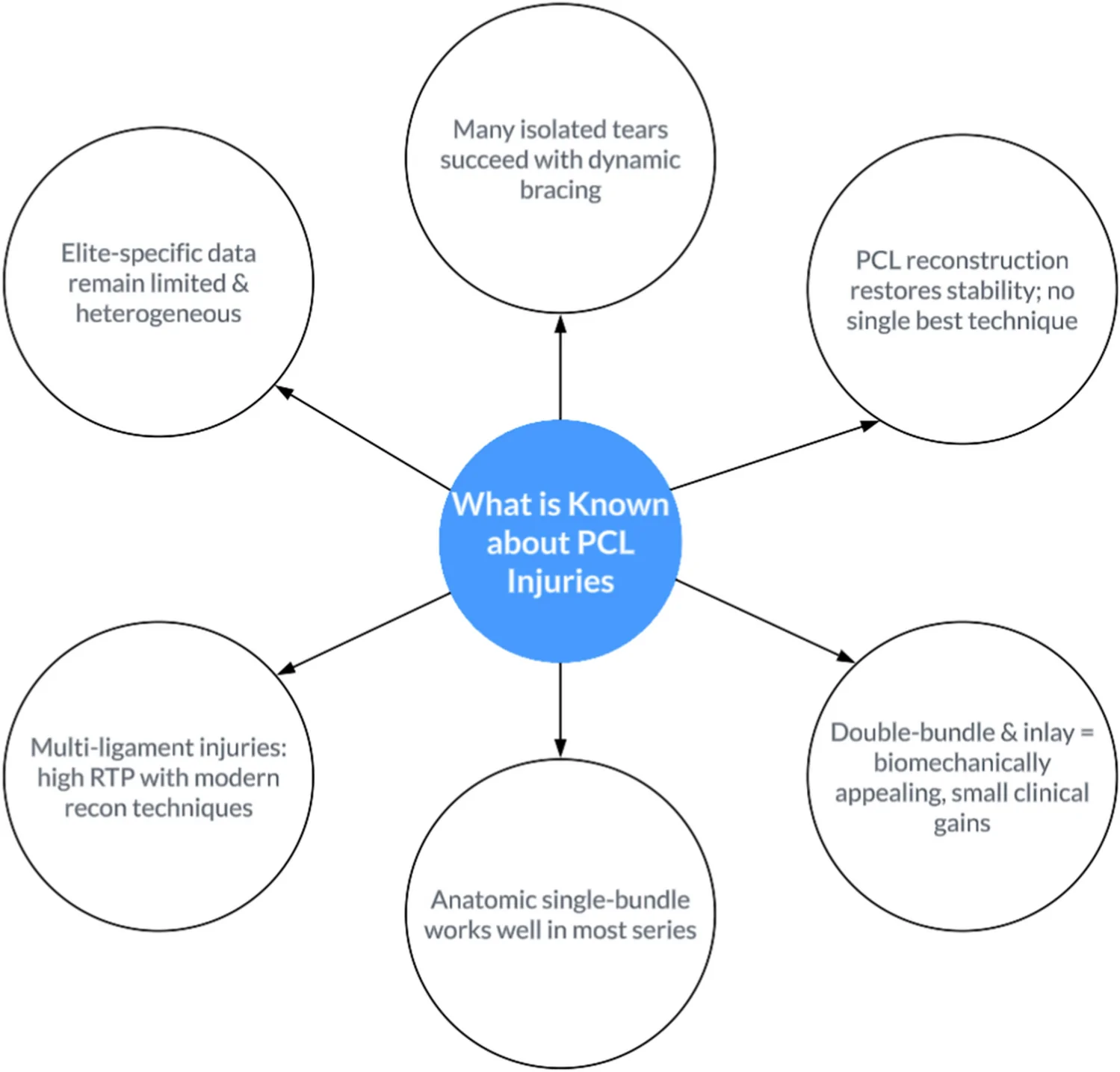
Established principles and current evidence in PCL injury management

Elite athletes impose extreme and often repetitive loads on the knee joint, particularly at high flexion angles and during deceleration, cutting, scrummaging, and grappling. These sport- and position-specific demands, combined with commercial and psychological pressure for rapid RTP, mean that decision-making in this group cannot simply extrapolate from recreational populations [4]. For the purposes of this review, “elite athlete” refers to athletes competing at professional level, full-time semi-professional level, or national/international representative level (including top-tier collegiate/scholarship programmes where sport is the primary performance focus). The aim of this narrative review is therefore to synthesise the current evidence on PCL reconstruction (PCLR) and non-operative management in athletes, focusing on graft choice, reconstruction strategy, fixation techniques, outcomes, and elite sport-specific considerations. An overview of established principles and current evidence in PCL injury management is shown in Fig. 2.

## Methods

**Fig. 3:**
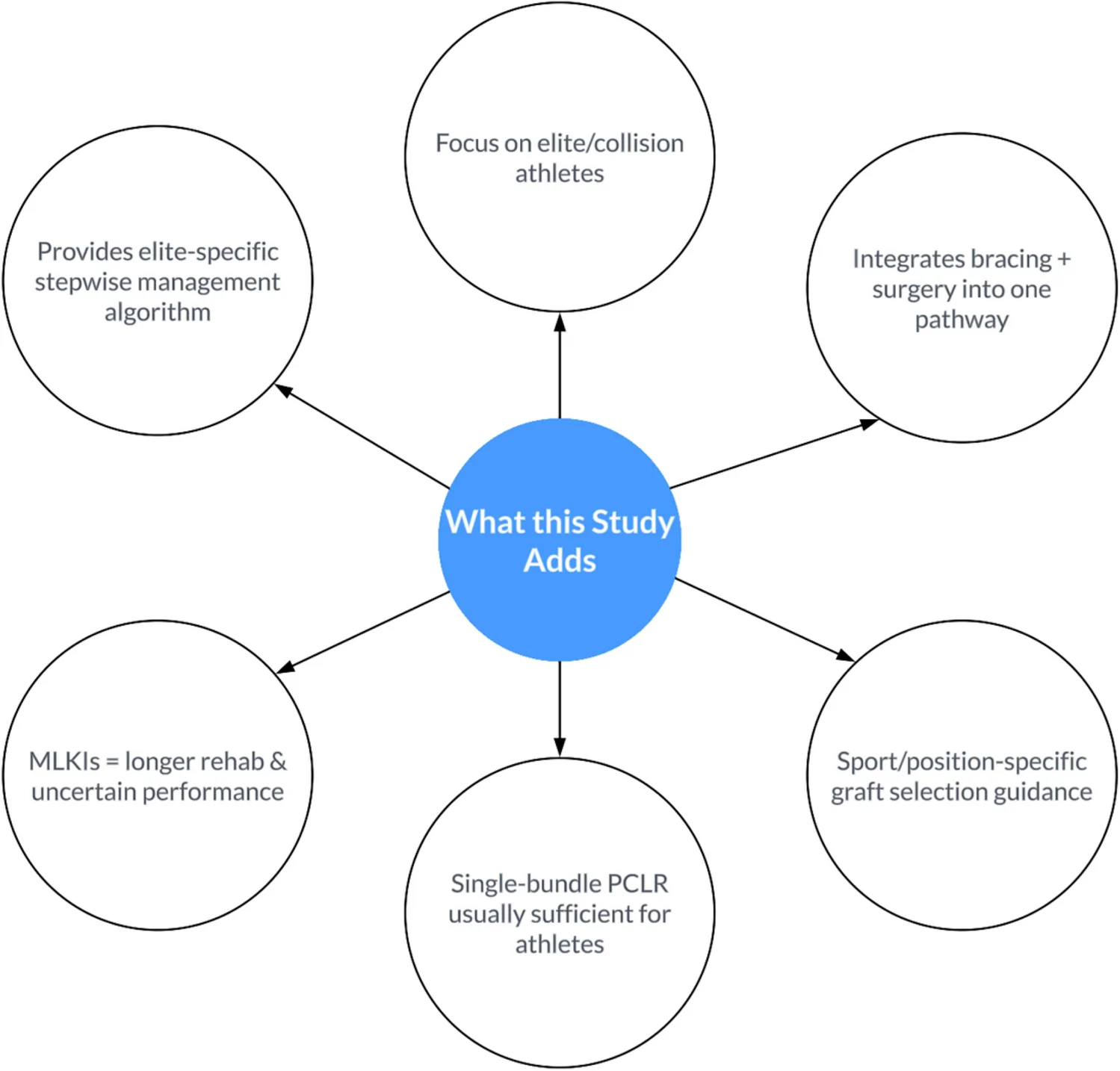
Novel contributions of this study to elite athlete PCL care

**Fig. 4:**
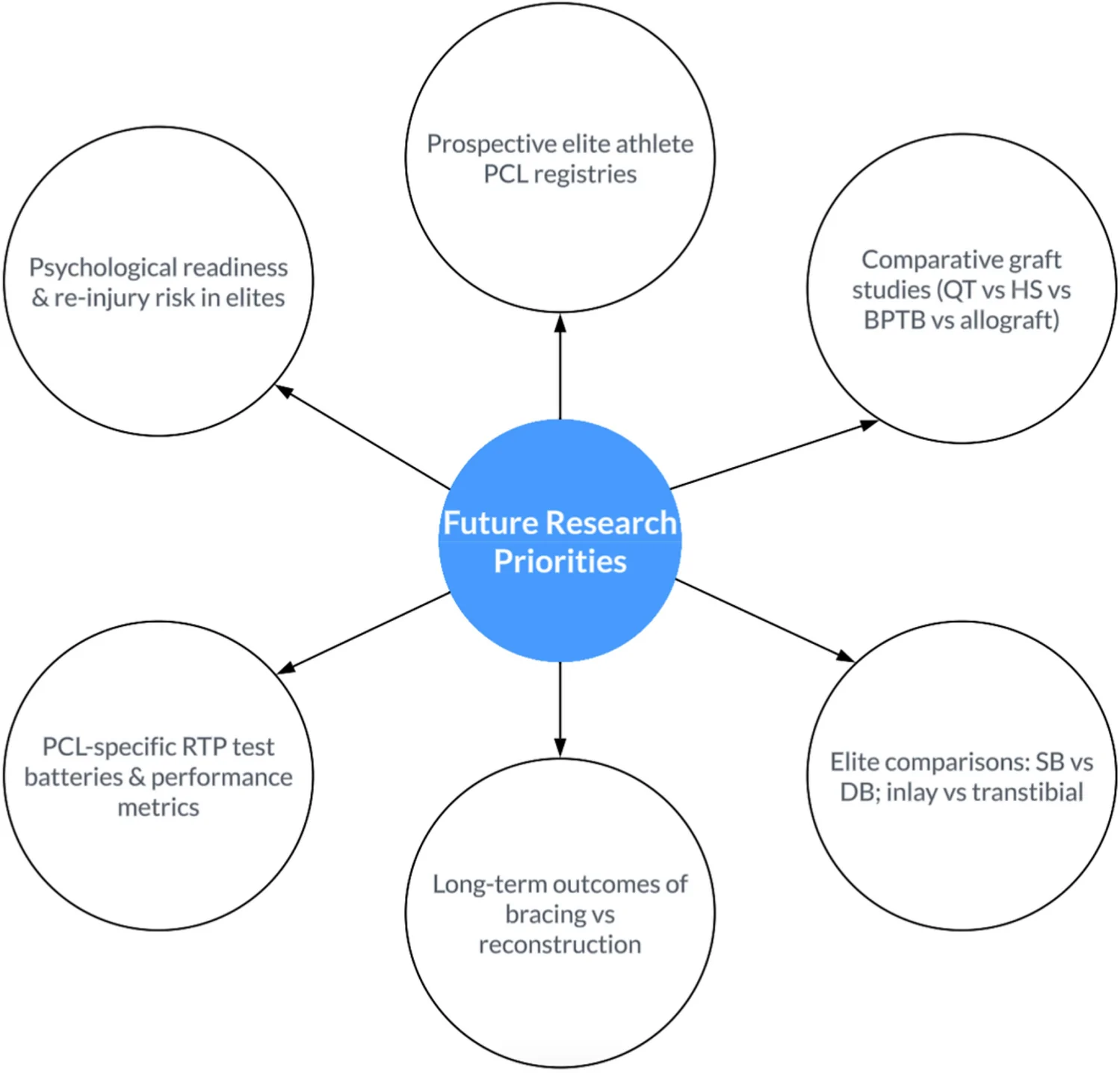
Future research directions and priorities for elite PCL injuries

A narrative review of the literature was performed using PubMed and Embase from database inception to April 2025. The search combined terms including “posterior cruciate ligament,” “PCL reconstruction,” “elite athlete,” “multi-ligament knee injury,” and “return to play,” with Boolean operators and appropriate MeSH/Emtree headings. Reference lists of included articles and relevant systematic reviews were additionally screened to identify eligible studies. Titles and abstracts were screened, and full texts were reviewed against predefined eligibility criteria. Included studies were clinical papers reporting RTP outcomes following management of PCL injuries (operative reconstruction/repair or non-operative rehabilitation), including isolated PCL injuries and PCL-based MLKIs; where reported, cohorts comprising professional, semi-professional, collegiate scholarship, or national/international-level athletes were prioritised. Exclusion criteria were cadaveric/biomechanical-only studies, technique descriptions without clinical RTP outcomes, imaging-only studies, non-athletic populations without stratified athletic outcomes, and studies in which the PCL was not a primary injury or PCL-related outcomes were not reported. 51 studies were included in the narrative synthesis. Given heterogeneity in injury patterns, interventions, and outcome definitions, PRISMA-style reporting and meta-analytic pooling were not undertaken. As a narrative review, the synthesis is subject to potential publication bias, selective outcome reporting, and over-representation of higher-profile athletic cohorts; these limitations were considered when interpreting comparative claims and generalisability across professional, semi-professional, and mixed-level samples. Overall risk of bias was appraised qualitatively for the narrative synthesis: the evidence base was predominantly Level III–IV (retrospective cohorts/case series) with moderate-high risk of selection and performance bias, heterogeneous interventions/outcomes, and inconsistent RTP definitions/reporting, limiting causal inference and pooled estimation (Figs. 3, 4).

### Epidemiology and Mechanism of Injury in Elite Sport

Epidemiological data from the UEFA Elite Club Injury Study suggest that PCL injuries in men’s professional football are rare, with an incidence of approximately 0.04 injuries per 1000 player-hours, much lower than for ACL or medial collateral ligament (MCL) injuries [5]. Most PCL injuries in this cohort result from contact mechanisms, including direct blows to the anterior tibia, collisions and tackles [3]. In contrast, non-contact pivoting mechanisms dominate for ACL injuries [6].

In rugby union, rugby league and American football, PCL injuries occur more frequently as part of MLKIs, especially in knee dislocations involving bi-cruciate rupture and disruption of one or both collateral ligaments and the posterolateral corner (PLC) [7]. However, collision-sport evidence is mixed and predominantly retrospective (Level III–IV evidence), so precise sport-specific incidence rates for PCL-based MLKIs remain poorly defined. These high-energy mechanisms reflect the collision nature of these sports and are associated with a higher risk of neurovascular compromise and long-term functional limitations, however, elite athlete–specific neurovascular incidence is rarely reported. Elite collision athletes with PCL-based MLKIs often face prolonged rehabilitation and uncertain return to pre-injury performance [8, 9].

### Non-operative Versus Operative Management in Elite Athletes

The management of acute isolated high-grade PCL tears in elite athletes remains controversial. Historically, many such injuries were treated non-operatively, based on the PCL’s healing capacity and acceptable outcomes in the general population [8]. More recent data in high-level athletes support this approach when combined with early diagnosis, dynamic bracing and targeted rehabilitation [9].

In a prospective cohort of professional and semi-professional athletes with acute isolated Hughston grade II–III PCL tears (Level III evidence), a structured protocol using a dynamic PCL brace and quadriceps-focused rehabilitation achieved ≥ 90% RTP to the same or higher level at 2 years, with mean time to RTP around four months [10]. At 5 years, the majority remained in competitive sport [10]. When viewed as a “truly elite” (professional-only) sensitivity analysis, interpretation is constrained as most studies do not report elite purity and rarely provide outcomes stratified by professional status. Therefore, it is important to interpret the ≥ 90% RTP figure as reflecting mixed high-level cohorts (professional/semi-professional) rather than a professional-only population. These findings suggest that early braced non-operative management is a reasonable first-line strategy for acute isolated PCL injuries in many elite athletes, particularly in-season and when concomitant meniscal or chondral pathology does not require surgery [11–13], while highlighting the need for future athlete registries to report professional-only subgroup outcomes.

Reconstruction is generally indicated in the elite setting for chronic symptomatic posterior instability despite structured conservative management, high-grade posterior laxity (often ≥ 10–12 mm on stress radiographs), PCL involvement in MLKIs or knee dislocations, and bony avulsions not amenable to fixation [14]. The presence of high-grade posterior laxity should alert the clinician to the possibility of concomitant injury to other ligaments, especially the posterolateral and posteromedial corners. In these cases, operative stabilisation is undertaken with the dual aims of restoring functional stability for elite performance and potentially mitigating long-term joint degeneration [3, 14]. A comparative summary of non-operative and operative strategies is provided in Table 1.

**Table 1:** Non-operative versus operative management of posterior cruciate ligament injuries in elite athletes

Domain	Structured braced non-operative management	Posterior cruciate ligament reconstruction
Typical indication in elite sport	Acute isolated grade II–III posterior cruciate ligament tears diagnosed within three to four weeks; no major concomitant meniscal or chondral pathology; in-season injuries where surgery would end the competitive season	Persistent symptomatic instability after structured braced rehabilitation; high-grade posterior laxity (greater than 10–12 mm on stress radiographs) in pivot or collision athletes; posterior cruciate ligament involvement in multi-ligament knee injuries or knee dislocation; irreparable bony avulsion or failed avulsion repair
Advantages	Avoids surgical risks and graft morbidity; faster early functional recovery; potential return to play within four to five months in carefully selected athletes	More predictable restoration of stability in high-grade laxity; allows comprehensive management of associated ligament, meniscal and chondral injuries; may better protect long-term joint health in unstable knees
Disadvantages	Risk of residual posterior laxity and subtle instability; possible long-term medial and patellofemoral compartment overload; outcomes highly dependent on brace compliance and rehabilitation quality	Longer total rehabilitation period (typically 9 to 12 months; up to 18 months in multi-ligament knee injuries); surgical complications and graft failure risk; donor-site morbidity with autografts, particularly in multi-ligament reconstructions
Elite return-to-play characteristics	Prospective athletic cohorts report more than 90% return to the same or higher level at medium-term follow-up for acute isolated injuries; best results in early-diagnosed, isolated tears with high brace compliance	Isolated posterior cruciate ligament reconstruction: high return-to-sport rates (approximately 70–90%) in active populations, with limited elite-specific data; multi-ligament injuries: recent elite series report approximately 80–90% return to play, but full return-to-performance is less frequent, especially in posterior cruciate ligament-based and bi-cruciate patterns

^a^*PCL* posterior cruciate ligament, *PCLR* posterior cruciate ligament reconstruction, *MLKI* multi-ligament knee injury

ᵇWhere exact *n*/*N* or precision estimates are not reported in the source studies, values are listed as NA

### Graft Choice in Elite Athletes

Several systematic reviews and comparative studies have evaluated graft choice in PCLR, but most include heterogeneous patient groups rather than elite athletes. Overall, hamstring autograft, bone–patellar tendon–bone (BPTB) autograft and quadriceps tendon (QT) autograft all produce acceptable stability and functional outcomes [15, 16]. However, elite athletes raise additional considerations such as sprint performance, deep-flexion kneeling demands, previous graft harvests and long-term donor-site morbidity [14].

Hamstring autograft (semitendinosus with or without gracilis) is widely used, particularly for transtibial PCLR. It offers high tensile strength, sufficient graft length for double-bundle (DB) constructs and technical familiarity [17]. In elite sprint-dependent athletes and wide football players, however, potential reductions in eccentric hamstring strength and sprint performance are a concern [18]. BPTB grafts provide bone-to-bone healing and high initial fixation strength but are associated with anterior knee pain, kneeling discomfort and risk of patellar tendinopathy, which may be problematic for rugby forwards, goalkeepers and athletes in kneeling martial arts [15].

Quadriceps tendon autograft has gained popularity for PCLR because it offers a large graft diameter with minimal donor-site morbidity and preserves both hamstrings and patellar tendon [19]. This makes QT particularly attractive for most elite athletes, especially those relying on high-speed running, repeated accelerations and kicking [19]. Additional autograft options such as peroneus longus and combined grafts may be considered in revision settings or when traditional graft sites have been exhausted [20]. Allograft is often reserved for MLKIs, older patients and revisions [21], as concerns remain regarding slower graft incorporation and potentially higher failure rates in younger, high-demand athletes [4, 15, 22]; Key graft options and elite-sport considerations are summarised in Table 2.

**Table 2:** Graft options for posterior cruciate ligament reconstruction in elite athletes

Graft type	Key advantages	Key disadvantages	Preferred indications in elite sport
Hamstring autograft (semitendinosus ± gracilis)	Long graft length suitable for single- or double-bundle constructs; high tensile strength; familiar harvest and fixation techniques	Potential reduction in eccentric hamstring strength; possible sprinting and cutting deficits; donor-site soreness or cramping in some athletes	Power and strength athletes without heavy sprint demands; situations requiring long or two-bundle grafts; revision cases when quadriceps tendon or bone-patellar tendon-bone grafts have already been harvested
Bone–Patellar Tendon–Bone (BPTB) autograft	Bone-to-bone healing with rigid fixation; high initial fixation strength; extensive experience from cruciate ligament surgery	Anterior knee pain and kneeling discomfort; risk of patellar tendinopathy or fracture; problematic in athletes who kneel frequently	Running and cutting athletes without kneeling demands; high-load sports where rigid fixation is prioritised; cases where previous hamstring or quadriceps tendon harvest limits other options
Quadriceps tendon (QT) autograft (± bone block)	Large graft diameter with excellent strength; minimal donor-site morbidity in most series; preserves both hamstrings and patellar tendon	Technical variation in harvest and learning curve; possible anterior thigh pain or temporary weakness	Most elite athletes, particularly speed-dependent players; footballers and rugby backs requiring preserved hamstrings; athletes with prior hamstring or BPTB harvest
Soft-tissue allograft (e.g. Achilles, tibialis, BPTB)	Avoids autograft donor-site morbidity; allows multiple ligament reconstruction in one setting; useful when autograft options are limited	Slower biological incorporation compared to autograft; potentially higher failure risk in young, high-demand athletes; availability and cost constraints in some health systems	Complex multi-ligament knee injuries requiring multiple grafts; older collision athletes or revision surgery; situations where autograft harvest is undesirable or unsafe

ᵃ*BPTB* bone–patellar tendon–bone, *QT* quadriceps tendon

### Single- Versus Double-Bundle Reconstruction

The native PCL comprises an anterolateral (AL) and posteromedial (PM) bundle that function in a flexion-dependent and reciprocal manner [3]. Biomechanical studies indicate that DB reconstruction most closely reproduces native knee kinematics and restores posterior stability throughout the range of motion [23, 24]. Nevertheless, clinical series and meta-analyses comparing single-bundle (SB) and DB PCLR show only small differences in posterior laxity on stress radiographs and no consistent, clinically meaningful advantages in patient-reported outcome measures (PROMs) such as International Knee Documentation Committee (IKDC) and Lysholm scores [25–27].

Several studies have reported that DB reconstruction produced a small improvement in stress radiograph side-to-side difference (pooled WMD with 95% CI) [28], but this incremental gain has not translated into demonstrably higher RTP rates or superior long-term function [24, 28]. In contrast, SB techniques are technically simpler, require less graft tissue and may be advantageous where multiple ligaments require reconstruction. There is currently no elite athlete-specific evidence to suggest that DB reconstruction leads to better performance or reduced re-injury risk compared with well-executed anatomic SB reconstruction.

In practice, surgeon familiarity with an anatomic SB technique, accurate tunnel placement on the native PCL footprint and robust fixation are likely to have a greater impact on outcomes than the number of bundles [3]. DB reconstruction may be considered in select cases such as large bony insertions, revision PCLR, or athletes with extreme deep-flexion demands, but its routine use in elite sport is not currently supported by clinical data [29]. SB versus DB reconstruction considerations are summarised in Table 3.

**Table 3:** Single- versus double-bundle posterior cruciate ligament reconstruction in elite athletes

Aspect	Single-bundle (SB) reconstruction	Double-bundle (DB) reconstruction
Biomechanical restoration	Restores posterior stability in mid-range flexion but is less anatomical than native posterior cruciate ligament bundles	Closer reproduction of native posterior cruciate ligament kinematics; better control of posterior translation across the flexion range in cadaveric studies
Clinical outcomes (patient-reported outcomes and laxity)	Good to excellent patient-reported outcomes in most series; small residual posterior laxity is common but often asymptomatic	Slightly reduced posterior translation (approximately 1–2 mm) on stress radiographs compared with single-bundle techniques; patient-reported outcomes generally similar at short- to mid-term follow-up
Technical considerations	Technically simpler and quicker; requires less graft tissue; easier tunnel placement and fixation, particularly in multi-ligament reconstructions	Technically more demanding with greater tunnel congestion; requires more graft tissue; increased risk of tunnel convergence in complex multi-ligament knee injuries
Rehabilitation implications	Standard posterior cruciate ligament rehabilitation protocols applicable; slightly fewer concerns about over-constraint in deep flexion	Similar rehabilitation principles; theoretical risk of overconstraint, but no clear evidence of different return-to-play timelines
Elite athlete-specific issues	High satisfactory when anatomic and well fixed; adequate for most elite athletes including footballers and many rugby players	May be considered in select athletes with extreme deep-flexion demands (e.g. front-row forwards, wrestlers); no proven superiority in return-to-play or performance over single-bundle techniques in elite cohorts
Current evidence	Comparative clinical studies/meta-analyses report similar PROMs and laxity *n*/RTP (RTP = return-to-play reported in study): Yoon 2019 [23]: *n* = 28; RTP not reported Chen 2025 [27]: *n* = 35; RTP not reported Lee 2017 [28] (4 RCTs): pooled *n* = 107; RTP not reported	Comparative clinical studies/meta-analyses show slightly less posterior translation but similar PROMs *n*/RTP (RTP = return-to-play reported in study): Yoon 2019 [23]: *n* = 36; RTP not reported Chen 2025 [27]: *n* = 26; RTP not reported Lee 2017 [28] (4 RCTs): pooled *n* = 108; RTP not reported

ᵃ*SB* single-bundle, *DB* double-bundle

ᵇPosterior translation values are reported as described in the cited comparative studies; where dispersion (SD/CI) is not provided, precision is NA

### Fixation Strategies and the Tibial Side Problem

The tibial side is widely regarded as the critical point in PCLR due to the so-called killer turn, where the graft bends sharply at the posterior tibial tunnel aperture and may be exposed to high local stresses and abrasion [15, 30]. The tibial inlay technique was developed to address this concern by fixing the graft directly to the posterior tibial cortex, thereby avoiding the killer turn [30]. Biomechanical studies demonstrate that inlay constructs reduce graft bending and may distribute load more evenly [30].

Despite these theoretical advantages, comparative series and reviews have not identified major differences in functional outcome scores, posterior laxity or osteoarthritis progression between tibial inlay and transtibial PCLR [10, 31]. Long-term follow-up studies of isolated PCLR show similar Lysholm, Tegner and IKDC scores for both techniques. As a result, many surgeons continue to favour transtibial approaches because they are less invasive, more familiar arthroscopically and compatible with remnant-preserving strategies [32].

Tibial inlay remains a valuable option in chronic PCL deficiency with significant attenuation, in revision PCLR where tunnel position or widening precludes a transtibial tunnel, and in cases where the posterior knee is already exposed for associated PLC reconstruction [33, 34]. On the femoral side, techniques that provide robust aperture or near-aperture fixation and minimise graft slippage during high-load rehabilitation are particularly important in elite athletes [15, 32].

### Remnant-Preserving and Stump-Sparing Techniques

Remnant-preserving PCLR techniques aim to maintain viable portions of the native PCL tibial stump and meniscofemoral ligaments, most commonly by preserving the AL bundle remnant and integrating the graft alongside retained fibres [35]. Potential advantages include a biological “pulley” effect at the killer turn, enhanced graft revascularisation, preservation of proprioceptive mechanoreceptors has also been proposed, but any clinically meaningful proprioceptive benefit remains speculative and is supported primarily by low-level evidence (Level IV) [36]. Prospective series of remnant-preserving PCLR using trans-septal posterior portals have reported good posterior stability, largely normal graft appearance on magnetic resonance imaging (MRI) and return to pre-injury sports levels in approximately 80–86% of patients [35].

Comparative studies suggest that remnant preservation may improve muscle torque and posterior stability relative to remnant-sacrificing techniques, although the evidence base remains limited and heterogeneous [35]. In the context of elite sport, the potential proprioceptive benefits and protective effect at the tibial bend are appealing, but no study has yet demonstrated superior RTP or return-to-performance (RTPe) outcomes specifically in elite athletes. Remnant-preserving PCLR is technically demanding and should be undertaken by surgeons experienced in advanced arthroscopic posterior knee surgery, particularly when deep flexion and rapid high-load rehabilitation are anticipated [37].

### Rehabilitation and Return-to-Play Criteria

Rehabilitation after PCL injury or reconstruction must balance protection of the healing ligament or graft against the detrimental effects of prolonged immobilisation and deconditioning [38]. Contemporary PCLR protocols typically permit early but protected range of motion, often 0–90° during the first 4–6 weeks, with close control of posterior tibial translation [39]. Isolated hamstring loading is avoided in the early phases, while quadriceps activation is encouraged with the tibia held in an anteriorly translated position [8].

Dynamic PCL bracing is commonly used for 3–6 months after both non-operative and operative treatment to maintain the tibia in a relatively anterior position during functional activities [3]. Progressive closed-chain strengthening, neuromuscular training, plyometrics, and sport-specific drills are introduced in a phased manner according to healing milestones and objective testing [40].

Objective RTP criteria should include near-symmetrical strength, satisfactory performance on functional hop and cutting tests, absence of effusion or instability symptoms, and adequate psychological readiness [41, 42]. Commonly used functional batteries include the single-leg hop for distance, triple hop, crossover hop, and 6-m timed hop, with targets of ≥ 95% limb symmetry on at least 3/4 hop tests (and ≥ 90% symmetry for isokinetic strength), alongside acceptable movement quality during landing/cutting tasks [43]. Although the evidence base is smaller than for ACL, psychological readiness (confidence and fear of re-injury) is increasingly recognised as a relevant determinant of RTS following PCL injury and reconstruction, and should be screened using ACL readiness instruments (e.g., ACL-RSI) [44]. Accordingly, RTS decision-making after PCL injury should include assessment of psychological barriers, as these factors may delay RTS despite satisfactory physical recovery and are potentially modifiable with targeted training and graded exposure [44]. Full-contact RTP after isolated PCLR is commonly delayed until 9–12 months, while MLKIs may require 12–18 months [45]. In contrast, braced non-operative management of acute isolated PCL injuries has been reported to facilitate earlier RTP (approximately 4–5 months) in carefully selected elite athletes, although athlete level and sport demands should be considered when extrapolating timelines.

Objective match-based key performance indicators (KPIs) (minutes played, match exposure and performance indicators) should be reported alongside RTP, as RTP reflects participation, whereas RTPe reflects return of competitive output [46]. Reliance on RTP alone may overestimate successful recovery, as athletes may return to competition but with reduced game time or a decrease in performance [47].

However, in PCL-based injuries, the evidence base for RTPe is very limited: most published series report time-to-RTP and/or return to the same or higher level of sport (often using activity scales such as Tegner) [10], rather than objective match output; and often minutes played/match exposure are not reported [10]. This contrasts with the ACL literature, where multiple studies quantify post-injury game time and performance, often demonstrating reduced participation and/or performance after return [48]; comparable high-quality RTPe datasets for isolated PCL injury or PCL reconstruction remain largely absent, limiting firm conclusions about post-return performance. A simplified pathway for elite athlete management is outlined in Table 4, and objective RTP criteria are summarised in Table 5.

**Table 4:** Operative versus non-operative rehabilitation pathways for PCL-based injuries in elite athletes

Step	Non-operative pathway (elite)	Operative pathway (elite)
1. Initial triage	Confirm isolated PCL versus PCL-based MLKI; baseline laxity/instability, effusion, ROM, associated meniscus/chondral injury	Same assessment; identify surgical indications (PCL-based MLKI/avulsion/instability or surgery-driving concomitant pathology)
2. Early protection	Dynamic PCL brace; reduce posterior tibial sag; activity modification	Post-op brace + protection; staged ROM; protect graft/repair and any collateral/PLC reconstructions
3. Rehab focus	Quadriceps-dominant strengthening; progressive neuromuscular control; gradual running then cutting/contact	Progressive ROM + strength (quad priority); staged running then cutting/contact; MLKI often slower progression
4. RTP criteria (objective)	No effusion/instability; full ROM; ≥ 90–95% strength symmetry; functional hop/cutting battery; psychological readiness	Same criteria plus stable exam/appropriate healing; often stricter/longer progression in MLKI
5. Typical timelines	Selected isolated PCL: sport return often reported around ~ 4–5 months (variable)	Isolated PCLR: ~ 9–12 months; PCL-based MLKI: ~ 12–18 months (pattern-dependent)
6. Return-to-performance (RTPe) monitoring	Minutes played/match exposure, starts/snap counts, performance versus baseline after RTP	Same monitoring after RTP

ᵃ*PCL* posterior cruciate ligament, *MLKI* multi-ligament knee injury, *RTP* return to play, *RTPe* return to performance, *ROM* range of motion, *PCLR* posterior cruciate ligament reconstruction

ᵇTimeline ranges are indicative and pattern-dependent, where study-level time data are not reported, values are NA

**Table 5:** Objective return-to-play criteria after PCL injury in elite athletes

Domain	Key RTP criteria	Purpose in elite athletes
Strength and power	≥ 90–95% limb symmetry index (quadriceps and hamstrings); restoration of sport-specific hamstring-quadriceps ratios; symmetrical jump and landing mechanics	Ensures adequate dynamic anterior support of the tibia and readiness for high-load deceleration, sprinting and contact
Neuromuscular control and functional performance	Near-symmetrical performance on combined hop, balance and agility tests (e.g. Y-balance, triple hop, cutting and change-of-direction drills)	Detected residual instability not seen on clinical exam and reflects readiness for chaotic, multiplanar sport demands
Sport-specific metrics	Completion of progressive running programme; sprint and GPS load metric within ≥ 90–95% of baseline and position norms; ability to perform position-specific skills without symptoms	Confirms readiness for maximal performance and high-speed loading relevant to competition
Clinical and psychological readiness	No effusion; no posterior sag; stable posterior drawer; satisfactory psychological readiness score (e.g. ACL-RSI-adapted)	Confirms joint stability and addresses psychological barriers that strongly influence RTP success

ᵃ*RTP* return to play

### Outcomes and Return to Play After PCLR

Isolated PCLR in active patients typically yields substantial improvements in posterior stability and PROMs, with many series reporting 70–90% good to excellent outcomes [10–13]. In a cohort of patients treated with arthroscopic AL bundle QT PCLR, posterior laxity improved by approximately 7–8 mm and 81% of patients returned to their pre-injury activity level [19].

In MLKIs that include the PCL, long-term outcomes have historically been guarded. Older open reconstruction series in elite athletes reported high rates of return to sport, but only about one-third regaining their exact pre-injury level, and a decrease in Tegner activity scores over time [49]. Delays beyond 4–6 weeks before definitive reconstruction were associated with worse satisfaction and lower activity levels.

More recent elite MLKI cohorts treated with modern anatomic reconstruction have demonstrated encouraging results. A series of 136 elite athletes treated with single-stage anatomic cruciate reconstruction and hybrid collateral/PLC management reported RTP to elite sport in 88% at a mean of 12.8 months [50]. ACL-based and PCL-based MLKIs had similar RTP rates, but PCL-based injuries required longer rehabilitation, with mean time to RTP 15.2 months for PCL-based versus 11.9 months for ACL-based injuries (*p* < 0.01) [50]. Bi-cruciate injuries required the longest recovery, with mean RTP time ~ 16.0 months versus ~ 12.4 months for uni-cruciate patterns (*p* < 0.05) [50]. In the National Football League, multi-ligament patterns involving the PCL and lateral structures have been linked to lower RTP and longer time to RTP compared with ACL/MCL injuries, underlining the impact of PCL-based MLKIs in collision sports [45].

### Knowledge Gaps and Future Directions

Significant knowledge gaps remain regarding PCL injury management in elite athletes. There is a paucity of prospective, sport-specific cohorts with clearly defined RTP and RTPe endpoints after PCLR or structured non-operative care. Comparisons of graft choice in elite populations—particularly QT versus hamstring versus BPTB versus allograft—are underpowered or absent. Going forward, there is the need for prospective elite registries both for graft choice and RTP outcomes.

Similarly, the relative merits of SB versus DB reconstruction and tibial inlay versus transtibial fixation have been explored biomechanically and in mixed clinical populations, but elite sport–specific data are lacking [51]. The long-term chondral and osteoarthritic sequelae of high-grade PCL laxity treated non-operatively in elite athletes remain unknown. Future research priorities include multi-centre registries of professional athletes with granular surgical, rehabilitation and performance data; prospective comparison of autograft and allograft in MLKIs involving the PCL; and development of validated PCL-specific RTP test batteries analogous to those now used after ACL reconstruction.

Long-term joint health after elite PCL injury is particularly under-reported. In a minimum 10-year follow-up of non-operatively treated acute isolated PCL injuries, Shelbourne et al. [13] reported moderate-to-severe radiographic osteoarthritis (OA) in 11% of patients, with generally preserved activity and subjective function; however, participants were not exclusively elite athletes, limiting direct extrapolation to professional sport [13]. Future prospective registries should pair imaging and symptoms with RTP KPIs to better capture the elite impact of PCL injury and its treatments.

## Conclusion

Elite PCL injuries range from partial tears to PCL-based MLKIs and knee dislocations. In selected acute isolated grade II–III tears diagnosed early, dynamic bracing plus quadriceps-focused rehabilitation can restore stability and allow earlier RTP with encouraging medium-term results, though evidence is largely from mixed professional/semi-professional cohorts [9, 10]. When instability persists, laxity is high, or the PCL is part of an MLKI, surgical stabilisation is often required. Across graft types, single- versus double-bundle constructs, and fixation strategies, clinical differences are generally small [15], so technique should be tailored to sport demands, anatomy, associated injuries, and prior graft harvests rather than a single “best” method. Successful elite RTP depends on correct indication, near-anatomic reconstruction with robust fixation, phased sport-specific rehabilitation, and objective, criteria-based RTP decisions that respect graft biology and performance pressures. Evidence remains limited in truly elite-only cohorts, and better sport-specific outcome and performance reporting is needed to define expectations and optimise return-to-play decision-making.

## Data Availability

No new data were generated or analysed in this narrative review. All data cited are from previously published literature and are publicly available through the referenced sources.
